# Predation by Bears Drives Senescence in Natural Populations of Salmon

**DOI:** 10.1371/journal.pone.0001286

**Published:** 2007-12-12

**Authors:** Stephanie M. Carlson, Ray Hilborn, Andrew P. Hendry, Thomas P. Quinn

**Affiliations:** 1 School of Aquatic and Fishery Sciences and Fisheries Research Institute, University of Washington, Seattle, Washington, United States of America; 2 Redpath Museum and Department of Biology, McGill University, Montréal, Québec, Canada; University of Bristol, United Kingdom

## Abstract

Classic evolutionary theory predicts that populations experiencing higher rates of environmentally caused (“extrinsic”) mortality should senesce more rapidly, but this theory usually neglects plausible relationships between an individual's senescent condition and its susceptibility to extrinsic mortality. We tested for the evolutionary importance of this condition dependence by comparing senescence rates among natural populations of sockeye salmon (*Oncorhynchus nerka*) subject to varying degrees of predation by brown bears (*Ursus arctos*). We related senescence rates in six populations to (1) the overall rate of extrinsic mortality, and (2) the degree of condition dependence in this mortality. Senescence rates were determined by modeling the mortality of individually-tagged breeding salmon at each site. The overall rate of extrinsic mortality was estimated as the long-term average of the annual percentage of salmon killed by bears. The degree of condition dependence was estimated as the extent to which bears killed salmon that exhibited varying degrees of senescence. We found that the degree of condition dependence in extrinsic mortality was very important in driving senescence: populations where bears selectively killed fish showing advanced senescence were those that senesced least rapidly. The overall rate of extrinsic mortality also contributed to among-population variation in senescence-but to a lesser extent. Condition-dependent susceptibility to extrinsic mortality should be incorporated more often into theoretical models and should be explicitly tested in natural populations.

## Introduction

Senescence is the physiological deterioration of older individuals, and is manifest as declines in survival probability or reproductive performance with increasing age. Senescence is thought to have its evolutionary origin in the action of environmentally-caused mortality–because this “extrinsic mortality” inevitably reduces the number of individuals reaching advanced ages. As a result, selection is weaker on genes that have negative effects late in life than on those that have negative effects early in life [1], [but see 2]. Under these conditions, mutations may accumulate that (a) improve early-life performance even at the expense of late-life performance (antagonistic pleiotropy, [3]), or (b) are unconditionally deleterious but are expressed only late in life (mutation accumulation, [4]). By extension, populations experiencing higher rates of extrinsic mortality should be under weaker selection against mutations with deleterious effects late in life, and should therefore evolve more rapid senescence [3], [4]. This classic evolutionary theory of senescence (classic ETS) has received broad support from studies showing that populations or species subject to higher rates of extrinsic mortality often show faster senescence [5]–[10]. Opposing results in some recent work, however, suggest that closer examination is warranted [11], [12; reviewed by 13], [Bibr pone.0001286-Bronikowski1] [14].

The classic ETS assumes that the state of senescence of an individual at a given time does not influence its susceptibility to *extrinsic* mortality at that time [3]. And yet, it seems quite likely that individuals showing advanced stages of senescence will be in poorer condition, and might therefore be more susceptible to extrinsic mortality (i.e., condition-dependent mortality). As an example, Trinidadian guppies (*Poecilia reticulata*) show reduced burst swimming speeds as they age, which should reduce their ability to avoid predators [12]. Such condition-dependent mortality may substantially alter evolutionary predictions. For example, Abrams [15] showed that higher extrinsic mortality could select for *deceased* senescence when senescence increase susceptibility to extrinsic mortality. Other recent theoretical [11] and empirical [12] work has further suggested that covariance between individual condition and extrinsic mortality can cause deviations from the classic ETS. To date, however, studies of senescence in nature have not evaluated the relative importance of extrinsic mortality *per se* (i.e., condition-independent) versus the degree of condition dependence in extrinsic mortality.

We assessed the relative importance of overall rates of extrinsic mortality versus the degree of condition dependence in extrinsic mortality by examining rates of senescence in breeding sockeye salmon (*Oncorhynchus nerka* Walbaum in Artedi, 1792) subject to predation by brown bears (*Ursus arctos* Linnaeus, 1758). In our study area, brown bears are by far the most important predator of breeding salmon (see below), and so they are a likely force driving the evolution of senescence. If extrinsic mortality *per se* is most important (i.e., the classic ETS), senescence should be slower in salmon populations experiencing lower rates of predation. If the condition dependence of extrinsic mortality is most important (henceforth the “condition-dependent ETS”), senescence should be slower in salmon populations where bears selectively kill fish showing advanced stages of senescence. This last prediction arises because such populations would experience greater direct selection against senescence [15]; i.e., individuals exhibiting less senescence at a given age would be more likely to survive in the face of extrinsic mortality.

Pacific salmon have several features that commend them to the study of senescence in nature. First, they show true senescence in the form of rapid physical deterioration from the time they start breeding until the time they die several weeks later [16], [17]. Second, they do not feed while breeding, and instead rely entirely on stored energy reserves. This “capital breeding” [18] sets up a trade-off between energy saved as somatic stores to fuel metabolism versus that invested into other aspects of reproduction (e.g., gonads and secondary sexual characters). Differential selection on the components of this trade-off can then cause adaptive variation in senescence [16], [19]. Third, the start of breeding reliably demarcates an appropriate physiological starting point for assessing senescence [16], [17].

In our study area, extrinsic mortality in breeding salmon is driven primarily by bear predation, which varies dramatically in intensity (“predation rate”) and the degree of condition dependence (“predator selectivity”). With regard to predation rate, bears kill up to 89% of the salmon breeding in some creeks but only 10% of those breeding in other creeks [20]. With regard to predator selectivity, bears generally prefer salmon showing little senescence because these fish have the highest energy density [21], [22], [23]. The problem for bears is that these “fresh” fish are more vigorous [24] and therefore more difficult for bears to catch. As a result, bears in small streams, where salmon are easy to catch, tend to kill salmon showing little senescence (i.e., newly arrived, energy-rich salmon at the beginning of their breeding lives) [21]. In contrast, bears in larger and more complex streams, where salmon are more difficult to catch, tend to kill salmon showing advanced senescence (i.e., energy-poor salmon at the end of their breeding lives) [21].

**Figure 1:**
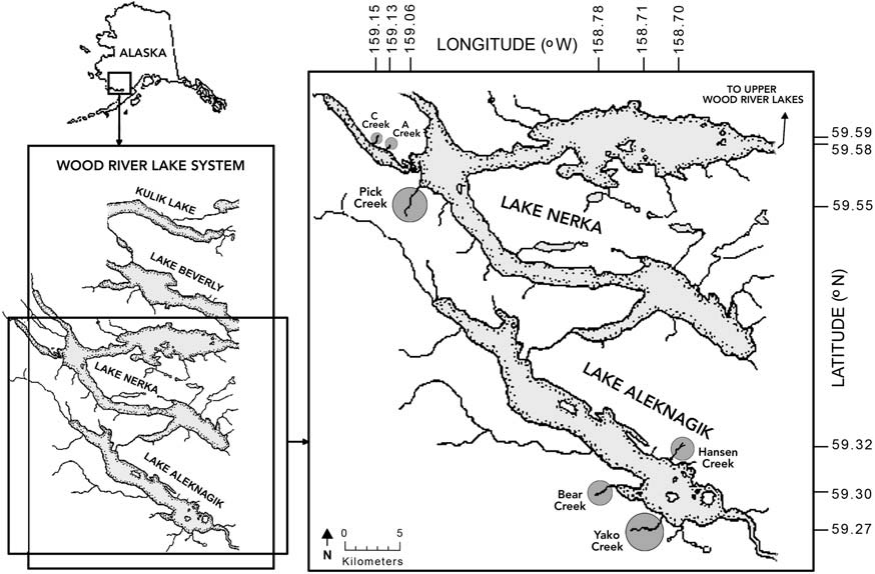
Sampling sites within the Wood River Lakes, southwest Alaska, USA.

Our goal was to determine whether variation in senescence was best explained by rates of extrinsic mortality (classic ETS) or the degree of condition dependence in extrinsic mortality (condition-dependent ETS). To compare these possibilities, we selected six Alaskan sockeye salmon populations (Figure 1) whose stream breeding habitats varied in ways that influence the intensity and selectivity of bear predation [20], [21] (Table 1). The six streams also differed in some environmental features that are unrelated to predation, such as stream temperature, distance from the ocean, and elevation (Table 2). Under the possibility that these other features influenced senescence rates, they too were evaluated. We suspect that any variation in senescence among populations is the result of adaptive divergence because (a) gene flow is limited among these populations (pair-wise F_STs_ based on microsatellites for three of our study populations range from 0.045 to 0.067, [25] and additional unpublished data), and (b) they show adaptive divergence in other life history and morphological traits [20].

**Table 1:** Some properties of the six streams and populations.

Creek	Creek Width (m)	Creek Depth (cm)	Reproductive lifespan (d)			Still alive at the end of the study
			mean±S.E. (N)			
			Senescent	Bear-killed	Other	N
A	1.4	10.0	12.65±0.23 (248)	4.36±0.12 (828)	6.02±0.55 (65)	294
Bear	5.1	19.3	13.43±0.31 (164)	8.40±0.19 (718)	7.29±0.81 (28)	19
C	2.1	10.0	12.9±0.24 (261)	7.36±0.17 (889)	5.57±0.75 (30)	518
Hansen	3.9	9.8	10.72±0.19 (226)	3.47±0.10 (753)	2.18±0.12 (258)	11
Pick	7.6	37.9	18.46±0.31 (276)	11.98±0.42 (263)	8.71±1.07 (31)	24
Yako	4.2	22.6	11.59±0.23 (152)	7.16±0.15 (771)	7.08±0.67 (24)	16

The mode of death “other” includes individuals that died owing to gull predation or that were stranded in areas of low water [32], [33]. The number of censored individuals in a given creek is equal to the number of individuals that did not die of senescence (i.e., the number of individuals killed by bears plus the number of individuals dying from other causes plus the number of fish still alive at the end of the study).

**Table 2:** Factors potentially driving inter-population variation in senescence rates.

Creek	Creek Temperature (°C) mean±S.E. (N)	Migration Distance (km)	Migration Elevation Gain (m)	Overall Predation Rate (% killed by bears) mean±S.E. (N)	Predator Selectivity For Newly Arrived Fish mean±S.E. (N)
A	5.93±0.13 (23)	106	23	88.42±6.51 (5)	0.165±0.003 (3)
Bear	9.28±0.13 (48)	44	10	29.80±3.09 (16)	0.067±0.004 (3)
C	7.21±0.19 (21)	106	23	78.72±11.10 (5)	0.087±0.002 (3)
Hansen	10.83±0.15 (48)	42	10	48.56±4.71 (18)	0.175±0.003 (3)
Pick	7.36±0.13 (48)	98	21	34.57±3.86 (17)	0.024±0.001 (3)
Yako	7.79±0.06 (48)	39	10	29.58±3.20 (15)	0.091±0.006 (3)

**Figure 2:**
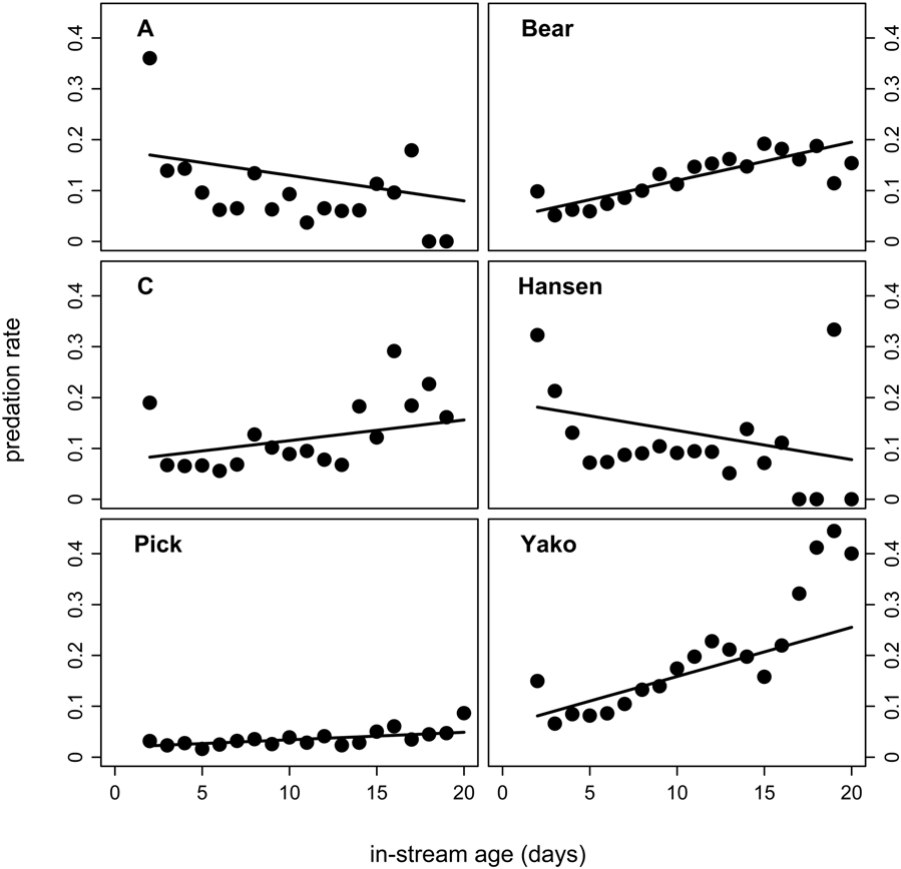
Population-specific predator selectivity for fish of different in-stream ages. Shown are proportions of the available fish of a given in-stream age (i.e., individuals that survived to day *d*) that are killed by bears in each creek. Our estimate of predator selectivity was the average of the predation rates on the first three days in the stream (i.e., the average of the first three points in each panel, see Electronic Supporting Information, Text S1). Note that the probability of being killed decreased within increasing in-stream age in Hansen and A creeks (i.e., bears killed salmon showing little senescence), but increased to varying degrees in Bear, Hansen, Pick, and Yako creeks (i.e., bears killed salmon showing more advanced senescence).

Creek temperatures were measured during the breeding period via hand-held thermometers or data loggers. Values are the average and SE across days (N). Migration distances were measured as the shortest straight-line water distance from the mouth of each focal creek to the ocean. Migration elevation gain was measured as meters above sea level for the lake into which the focal creek drains [44]. Predation rate represents the average of the yearly percent of breeding salmon killed by bears. The standard error represents the among year variation in the percent of salmon killed by bears. Predator selectivity for salmon showing little senescence represents the average (± SE) of the predicted predation rate across the first full three days in-stream (i.e., the average of the first three points presented in Figure 2).

Our analyses were based on two data sets. The first was used to estimate predation rates indicative of overall rates of extrinsic mortality, and was therefore applicable to testing the classic ETS. This data set was based on annual surveys that estimated the numbers of breeding salmon in each creek, as well as the proportion of these fish killed by bears. The second data set was used to estimate predator selectivity, and was therefore applicable to testing the condition-dependent ETS. This data set was based on 6,867 individually-tagged breeding salmon, including at least two years of data from each of the six creeks. Tagged fish were monitored from the day they entered the creek (start of breeding) until the day they died, an interval that defined their “reproductive lifespan” (Table 1). We also recorded the mode of death for each individual: senescent (*n* = 1,327), killed by bears (*n* = 4,222), or other (i.e., gull-killed or stranded in areas of low water, *n* = 436; Table 1). This individual-based data set was also used to estimate senescence rates in each population, which were then compared to the estimates of predation rate, predator selectivity, and other environmental variables.

## Results

The six populations differed markedly in predation rate, estimated as the average annual percentage of breeding salmon killed by bears (Table 2, see also Materials and Methods). They also differed in predator selectivity, estimated as the average predicted predation rate for individual salmon during their first three days breeding in a stream (Table 2). Note that our index of predator selectivity is based on salmon showing little senescence, whereas our predictions and interpretations are often based on selectivity for salmon showing advanced senescence. The reason for this apparent disconnect is that the two indices are inversely related and, although interpretations are more straightforward for the latter index, the former index can be estimated with much greater precision (see Materials and Methods and Electronic Supporting Information, Text S1).

Predation rate was lowest for Bear, Pick, and Yako creeks, intermediate for Hansen Creek, and highest for “A” and “C” creeks (Table 2). Based on this variation, the classic ETS would predict that senescence should be slowest in Bear, Pick, and Yako creeks, intermediate in Hansen Creek, and fastest in A and C creeks. On the other hand, predator selectivity for salmon showing little senescence was lowest for Pick Creek, intermediate for Bear, C, and Yako creeks, and highest for Hansen and A creeks (Table 2; Figure 2). Based on this variation, the condition-dependent ETS would predict that senescence should be slowest in Pick Creek, intermediate in Bear, C, and Yako creeks, and fastest in Hansen and A creeks.

We first confirmed that our populations manifest true senescence. We did so by using the Weibull distribution to model the probability of dying in a particular time interval given survival to that interval (“hazard function”) [6], [7], [26], [27]. This distribution is described by two parameters: *α* (shape of the hazard function) and *λ* (magnitude of hazard for a given shape of the function) [28]. When *α*>1, hazard increases with age and senescence is present [6], [27], [29]. We therefore compared the fit of a model in which *α* = 1 to models in which *α* was estimated from the data.

Comparisons between alternative senescence models (Table 3) yielded the following conclusions. First, our populations manifest true senescence–because models II-VII, in which *α* was estimated to be greater than unity (senescence), fit the data much better than did model I, in which *α* was set to unity (no senescence). Second, late breeders senesced more rapidly than early breeders within a given stream–because models III-VII, which included day-of-entry parameters (see Materials and Methods), always fit the data much better than did models I-II, which did not include these parameters. Third, senescence rates varied dramatically among the populations–because models V-VII, in which *α* and/or *λ* parameters varied among populations, always fit the data much better than did models I-IV, in which these parameters did not vary among populations.

**Table 3:** Candidate models explaining variation in senescence.

General Model Structure	NLL	No. Parameters	AIC	ΔAIC
I. *α* = 1, same *λ* for all creeks	6,211.50	1	12,425.00	3,669.59
II. Same *α* and *λ* for all creeks	4,807.23	2	9,618.47	863.06
III. Same day of entry (*b*), *α*, *λ* parameter for all creeks	4,698.21	3	9,402.42	647.00
IV. Different *b* for each creek, same *α* and *λ*	4,684.57	7	9,383.14	627.72
V. Different *b* and *α* parameters for each creek, same *λ*	4,382.78	13	8,791.56	36.15
VI. Different *b* and *λ* parameters for each creek, same *α*	4,375.23	13	8,776.46	21.05
VII. Different *b*, *α*, and *λ* parameters for each creek	4,359.71	18	8,755.41	0

We used the Weibull model [28] to estimate senescence rates. The Weibull model has two parameters that define the hazard function: *α*, which represents the shape of the hazard function, and *λ*, which represents the magnitude of the hazard given the shape of the function. We also included a day of entry parameter, *b*, because previous work has shown that early breeding salmon senesce slower than late breeding salmon [16]. Listed is the general model structure, the negative log-likelihood (NLL), the number of parameters (No. parameters), Akaike's Information Criterion (AIC), and the ΔAIC (relative to the best model, model VII). See Table S1 of the Electronic Supporting Information for explicit formulae for each of the above models.

Model VII, in which both *α* and *λ* varied among the populations, was the best model (Table 3) and its likelihood function was:
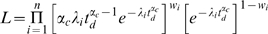
(1)where *α_c_* represents the *α* parameter in the *c* ^th^ creek, *λ_i_* is determined as in equation 10 (see Materials and Methods), *t_d_* represents the age at death (in days) of the *i* ^th^ individual, and *w_i_* represents the censoring indicator (see Materials and Methods). Likelihood functions for all seven models are presented in the Electronic Supporting Information (Table S1).

**Figure 3:**
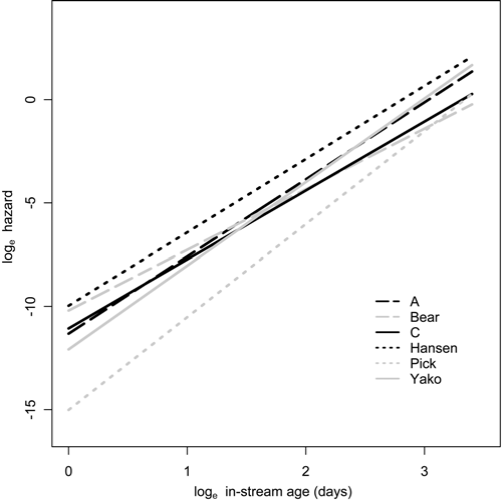
Hazard. Population-specific hazard functions (y-axis) plotted against in-stream age (x-axis). These functions are based on a mean day of entry and on population-specific day of entry parameters. The higher the hazard for a given in-stream age, the greater the senescence rate at that age. The slopes of these lines represent variation in the shape of the hazard function (*α*) and the elevation of the lines represent variation in their magnitude given the shape (*λ*).

**Figure 4:**
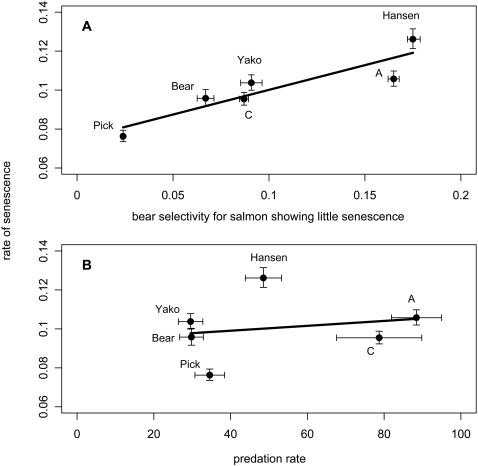
Rate of aging. Senescence rates (*ω*±95% confidence intervals generated from likelihood profiles, [42]) plotted against (A) predator selectivity for salmon that show little senescence (± SE across the first three days in the stream) and (B) predation rate (± SE across years). For both plots, the line represents the predicted senescence rates from an ordinary-least squares regression.

Age-specific (i.e., with respect to in-stream age) hazards based on model VII for individual *i* at time *t* (in days) were calculated as:
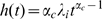
(2)Parameter values from this model were used in generating population-specific hazards (Figure 3), and in calculating senescence rates for each population (Table 4). Here we focus on Ricklefs' [26] shape-adjusted index of the rate of senescence (*ω*, see equation 6 in Materials and Methods), which revealed that senescence was slowest in Pick Creek, intermediate in C, Bear, Yako, A creeks, and fastest in Hansen Creek (Table 4; Figure 4). Although we focus on model VII because it was the best model, parameter estimates from model V (only *α* varies among populations) or model VI (only *λ* varies among populations) yielded similar conclusions (Electronic Supporting Information, Table S2). In short, our conclusions are not dependent on the particular senescence rate metric.

**Table 4:** Parameter estimates for the best model.

Population	*α*	*λ*	*ω*
A	4.73	2.57E-06	0.106 (0.102 to 0.110)
Bear	3.93	9.45E-06	0.096 (0.092 to 0.100)
C	4.33	3.61E-06	0.095 (0.092 to 0.099)
Hansen	4.55	1.03E-05	0.126 (0.121 to 0.131)
Pick	5.50	5.48E-08	0.076 (0.074 to 0.079)
Yako	5.05	1.12E-06	0.104 (0.100 to 0.108)

Parameter estimates for *α*, *λ*, and *ω* (95% confidence bounds in parentheses based on likelihood profiles, [42]) derived from the best model (i.e., model VII, Table 3) for explaining senescence variation. The *α* parameter represents the shape of the Weibull hazard function, *λ* represents the magnitude of the Weibull hazard given the shape of the function, and *ω* is a derived parameter that provides a shape-adjusted index of the rate of senescence [26].

Comparisons of potential factors explaining inter-population variation in senescence (Table 5) yielded the following conclusions (note that models here, as opposed to the above, are referred to by Arabic numerals). First, predator selectivity was more important than predation rate in explaining variation in senescence–because model 5 (predator selectivity) fit the data much better than did model 1 (predation rate). The best model, however, included predation rate, predator selectivity, and their two-way interaction (model 7). In this model, (a) populations where bears selectively killed salmon showing the least senescence (predator selectivity) were those that showed more rapid senescence (Figure 4A), (b) populations where bears killed a larger percentage of salmon (predation rate) were those that showed more rapid senescence (Figure 4B), and (c) the effect of predation rate was greatest when predator selectivity for salmon showing little senescence was highest.

**Table 5:** Candidate models for explaining inter-population variation in senescence.

	General Model Structure	NLL	No. Parameters	AIC	Δ AIC
1.	*ω* = *β*_0_+(*β*_1×_predation rate)	−16.84988	2	−27.70	15.96
2.	*ω* = *β*_0_+(*β*_1_×elevation gained during the migration)	−17.50571	2	−29.01	15.30
3.	*ω* = *β*_0_+(*β*_1_×migration distance)	−17.56752	2	−29.14	15.24
4.	*ω* = *β*_0_+(*β*_1_×water temperature)	−17.70858	2	−29.42	15.10
5.	*ω* = *β*_0_+(*β*_1_×predator selectivity)	−21.84695	2	−37.69	10.96
6.	*ω* = *β*_0_+(*β*_1_×predation rate)+(*β*_2_×predator selectivity)	−24.7532	3	−41.51	8.06
7.	*ω* = *β*_0_+(*β*_1_×predation rate)+(*β*_2_×predator selectivity)	−32.80872	4	−55.62	0
	+(*β*_3_×predation rate×predator selectivity)				

For each model, the shape-adjusted index of the rate of senescence (*ω*) was regressed against the factors listed under the general model structure. In each case, the betas represent the estimated parameters (*β*_0_ represents the intercept coefficient and *β*_ 1_, *β*_ 2_, and *β*_ 3_ represent slope coefficients). We have also listed the negative log-likelihood (NLL), the number of parameters (No. parameters), Akaike's Information Criterion (AIC), and the ΔAIC (relative to the best model, model 7).

We also tested whether inter-population variation in senescence was related to environmental conditions that do not influence predation but might influence senescence in other ways, such as through changes in energy depletion (Table 2). Here, a model including elevation gained during the migration (model 2), migration distance (model 3), or water temperature (model 4), received only weak support (Table 5). These models (models 2–4, Table 5) never fit the data better than models including predator selectivity (models 5–7, Table 5), suggesting that environmental factors other than predation did not drive the among-population variation in senescence rates.

## Discussion

Among site variation in predation by bears was strongly associated with rates of senescence in natural populations of breeding sockeye salmon. We tested two possible explanations for this pattern. First, the classic evolutionary theory of senescence would predict a positive association between overall rates of extrinsic mortality and rates of senescence [3]. Second, the condition-dependent theory of senescence would predict a positive association between predator selectivity for fish showing little senescence and the rate of senescence [11], [15]. We found strong support for the condition-dependent prediction and some additional support for the classic prediction, although only when condition dependence was also considered (Table 5). Interestingly, senescence rates further appear influenced by an interaction between the two aspects of predation: overall rates of extrinsic mortality were most important when predators were most selective for newly-arrived salmon showing little senescence. We interpret these among-population correlations as evidence of adaptive genetic divergence in response to local bear predation. We now consider two alternatives, which are not mutually exclusive: variation in senescence might be (a) driven by environmental factors other than predation, and (b) the result of phenotypic plasticity rather than genetic divergence.

The first possibility is easy to discount given the lack of evidence for any role of environmental factors other than predation. First, the close geographic proximity of our study populations (Figure 1) leads to minimal variation in climate, day length, parasite infection (nematode, *Philonema oncorhynchi*, [30]), and water chemistry. Second, the timing of transition from the ocean to fresh water does not differ appreciably among these populations [31]. Third, environmental factors that do vary among populations (e.g., water temperature, elevation gained during the migration, and migration distance; Table 2) are not correlated with senescence (Table 5). Fourth, other forms of extrinsic mortality, such as predation by gulls or “stranding” in shallow water [32], [33], accounted for relatively few of the deaths (6.3%, Table 1). Interestingly, the one environmental factor (water depth, Table 1) that did correlate to some extent with senescence (*r^2^* = 0.58, *p* = 0.081) is the exception that proves the rule. Specifically, shallower water makes it easier for bears to catch fish in small streams [20], and so it is at these sites that bears can express selectivity for salmon that show little senescence [21]. In short, environmental factors other than those related to bear predation are unlikely to have driven the among-population variation in senescence rates.

The second possibility, phenotypic plasticity rather than adaptive divergence, cannot be refuted by direct evidence. For example, common-garden experiments are too daunting for salmon owing to their large body size (∼2–4 kg), late age at maturity (∼4–6 years), and need for flowing water. Moreover, controlled experiments would be inappropriate for our study because senescence under such conditions would not reflect senescence in nature. Reciprocal transplant experiments might be another option, but these are thwarted by the tendency of displaced adults to depart for their natal sites [34]. In the absence of direct evidence, we turn to indirect arguments. Here we first note that adaptive divergence seems plausible given that our study populations show (a) strong natal homing that would limit the homogenizing role of gene flow ([25] and additional unpublished data), and (b) evidence that other traits have adapted to bear predation [20]. We next note that plastic effects of bear predation on senescence are unlikely to be strong. Bear activities probably do stress fish that are not killed, but the amount of time that an individual fish would be disturbed by a bear is relatively small and unlikely to materially influence senescence. In short, we have no reason to suspect that the observed variation in senescence is anything other than adaptive genetic divergence in response to selection imposed by bear predation.

### Implications

Why do some studies provide strong support for the classic ETS [5]–[10], whereas others do not [11], [12]? Among the several possibilities, our results yield insight into the potential role of condition-dependent extrinsic mortality. We first suggest that condition dependence can sometimes be the primary determinant of variation in senescence rates, as it was in our study. We next suggest that the importance of overall rates of extrinsic mortality may depend on how this aspect of bear predation correlates with condition-dependent mortality. In some systems, these two aspects of predation may be closely correlated, and so reinforce each other in driving the evolution of senescence. In other systems, such as ours, the correlation may be weaker, and associations between senescence and extrinsic mortality rates may be difficult to detect (because condition-dependent predation is more important). Indeed, we were only able to infer a role for rates of extrinsic mortality (predation rate) after also accounting for the role of condition-dependent mortality (predator selectivity). Jointly considering these two aspects of extrinsic mortality further revealed an interesting interaction: extrinsic mortality was only important when condition dependence was strong. Further empirical data from natural systems, combined with theoretical models incorporating condition-dependent extrinsic mortality, are needed to test the above ideas and to better understand recent exceptions to the classic ETS.

## Materials and Methods

### Field sampling

**Figure 5:**
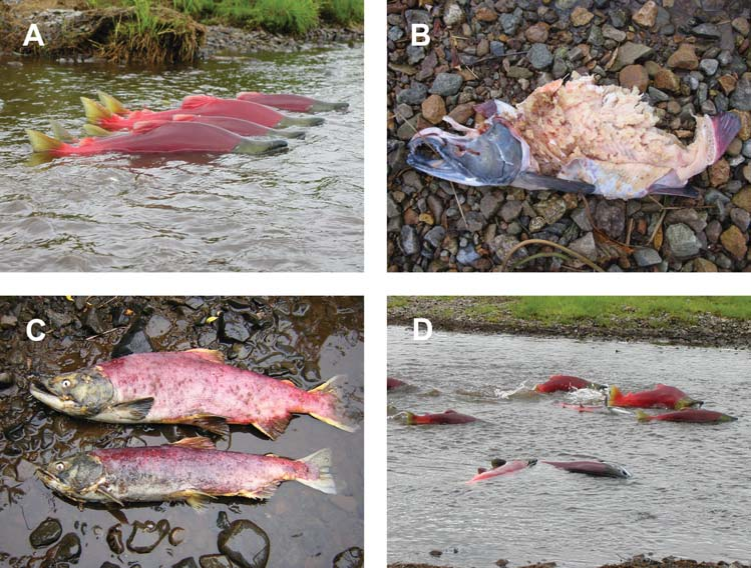
Photographs showing breeding sockeye salmon in various states. Panel A shows newly arrived sockeye salmon that show little senescence. Note their bright red coloration. Panel B shows a bear-killed male salmon. Panel C shows a senescent male (top) and female (bottom) salmon. Note their frayed fins, drab coloration, and general emaciated appearance relative to the newly arrived fish. Panel D shows salmon that have stranded in an area of low water (bottom left corner of panel D). Photographs by Ranae Holland (A), Stephanie Carlson (B,C), and Neala Kendall (D).

Sockeye salmon in our study area return from the ocean in late June and then shoal in lakes adjacent to their natal creek for a few weeks until maturation is complete. Mature salmon then enter the creeks and start breeding almost immediately [19], [35]. To ensure that we monitored individuals from when they started breeding, we captured fish prior to creek entry (using seine nets at the mouth of the creek) or immediately after creek entry (using landing nets). Each captured fish was tagged with an individually-coded, external disk tag (3 cm diameter), a procedure that does not have noticeable effects on survival or breeding behavior [19], [35]. We then used daily stream surveys to determine the start of breeding for each fish (the day it entered the creek), and whether it was still alive in the creek on each subsequent day. These data can be collected with high confidence and precision because the water in all of these creeks is clear and shallow enough to spot essentially all of the breeding fish and read their tags each day (see Figure 5A).

The date of death for each fish was assumed to occur the day after it was last observed alive. In many cases, this date coincided with the recovery of that individual's carcass, which thus confirmed the date of death. In other cases, an individual's carcass was never recovered, almost certainly because it had been killed by a bear who then carried it away from the stream (see below). In these cases, it is still safe to assume that the fish died within a day of when it was last seen alive because living fish were rarely missed when surveying the stream. Reproductive lifespan was calculated for each tagged fish as the number of days between when it entered the creek and when it died.

The carcasses of recently-dead salmon manifest obvious indicators of the mode of death [16], [19], [35]–[38]. *Bear-killed* salmon have large wounds and pieces of missing flesh (Figure 5B). *Senescent* salmon are emaciated, have frayed fins and rough skin, and lack penetrating wounds (Figure 5C). *Stranded* salmon (those that get caught in shallow water and suffocate [32], [33]) lack penetrating wounds, show little signs of senescence, and are found in very shallow areas of the creek (Figure 5D). *Gull-killed* salmon have distinctive radial chiseling wounds that penetrate the body cavity near the gill plates, vent, or pectoral fins. Based on these criteria, the mode of death was assigned to each tagged fish whose carcass was recovered.

We also assigned the bear-killed mode of death to breeding fish whose carcasses were not found during our surveys. As noted above, these fish were almost certainly killed by bears and carried out of sight into the riparian zone [39], [40]. Very few of the missing fish would have died of other causes because the creeks have almost no areas where such carcasses could remain undiscovered during our surveys. Furthermore, previous work has demonstrated that the reproductive lifespans and body lengths of bear-killed fish and “missing” fish are similar and clearly different from those of senescent fish ([37] and additional unpublished data).

Data for Bear and Yako creeks were collected by SMC (Bear: 2003, *n* = 387; 2004, *n* = 542; Yako: 2003, *n* = 364; 2004, *n* = 599). Data for A and C creeks were collected by RH (A: 1998, *n* = 318; 2001, *n* = 225; 2004, *n* = 453; 2005, *n* = 439; C: 1998, *n* = 595; 2001, *n* = 381; 2004, *n* = 422; 2005, *n* = 300). Data for Pick Creek were collected by APH (1995, *n* = 247; 1996, *n* = 347). Data for Hansen Creek were collected by TPQ (1999, *n* = 126; 2000, *n* = 174; 2001, *n* = 173; 2002, *n* = 168; 2003, *n* = 205; 2004, *n* = 161; 2005, *n* = 241). The data for all creeks and years are directly comparable because the methods were identical–all primary investigators were trained by the same person (TPQ). Moreover, precautions were taken to ensure that exactly the same methods were maintained across years and streams. For example, APH and SMC repeatedly worked with TPQ on Hansen Creek, and APH repeatedly worked with RH on A and C creeks. The various other field personnel spent time on multiple creeks with multiple investigators, which further minimized the possibility of observer-driven variation among creeks.

### Predation rate (extrinsic mortality rate)

Previous work has shown that the annual predation rate on breeding salmon in a creek (percentage of all breeding adults killed by bears) can be reliably estimated based on a single survey during the peak of the breeding season [20]. This estimate is obtained as the average of two proportions: (a) the cumulative number of salmon killed by bears divided by the cumulative number of all dead salmon, and (b) the cumulative number of salmon killed by bears divided by the sum of the total number of live salmon on the day of the survey plus the cumulative number of dead salmon (Electronic Supporting Information, Figure S1A). This method was validated for our study by reference to a population where the mode of death is determined for all breeding salmon. In short, the single-survey method described above did a very good job of estimating the total proportion of fish actually killed by bears (Electronic Supporting Information, Figure S1B). Our subsequent analyses were then based on the average of the five to eighteen annual estimates of predation rate in a given stream (Table 2).

### Predator selectivity (condition-dependent mortality)

Newly-mature sockeye salmon usually enter a given creek over a period of 2 to 4 weeks [19]. Each individual may then live for another 1 to 3 weeks before dying of senescence–if it does not succumb earlier to predation or stranding (Table 1). Thus, for a period of several weeks, bears foraging on any given day are presented with a range of salmon of different “in-stream ages.” These ages are the number of days since an individual entered the creek, and are strongly indicative of its state of senescence: an individual shows more signs of senescence as it ages. We then estimated the probability of an individual being killed as a function of its in-stream age, given survival up to that age. In other words, we modeled the probability of being killed at each in-stream age given the total number of fish of that in-stream age that were available to the bears. Details of the method are provided in Gende et al. [21], and its application to the present data is described in the Electronic Supporting Information (Text S1).

To estimate predator selectivity, we used the results of the above modeling procedure to calculate an overall index of the degree to which predators killed fish showing *little* senescence. This index was calculated for each creek as the average of the age-specific (i.e., with respect to in-stream age) predation rates across the first three full days each fish was present in the stream (see also Electronic Supporting Information, Text S1, and Table 3). We also generated a second index of selectivity: the degree to which bears killed fish showing *little* senescence (as above) divided by the degree to which bears killed fish showing *advanced* senescence (average of the age-specific predation rates across the three oldest in-stream ages achieved in that stream). Although the two indices are highly correlated among streams (*r* ^2^ = 0.82), we here focus on the first index. One reason is that it can be estimated with much greater confidence than the second index owing to the many more fish showing little senescence (essentially all fish that enter the stream) than showing advanced senescence (only the few that survive to very advanced ages) [21]. Another reason is that predation on fish of advanced in-stream ages may be influenced by the evolution of senescence–if senescence influences susceptibility to predation. This would be a disadvantage because we are here seeking to compare predator selectivity among streams without any confounding influence of variation in senescence. Thus, note that although we often phrase our predictions and interpretations in the easier-to-understand context of selectivity for salmon showing advanced senescence, our index is of selectivity for salmon showing little senescence.

### Senescence rates

Senescence rates were evaluated by modeling survival probabilities with respect to in-stream age using the Weibull model [28] according to the convention of several recent studies [6], [7], [16]. This model is particularly appropriate because the rate of senescence parameter (*ω*, described below) is independent of the rate of extrinsic mortality [27]. The Weibull model has two parameters that define the hazard function (the probability of dying in a particular time interval given that the individual survived up to that time interval): *α*, which determines the shape of the hazard function, and *λ*, which determines the magnitude of the hazard given the shape of the function.

In the Weibull model, the *survivor function* is the proportion of individuals from the initial cohort that is still alive at some future time, *t* [28]:
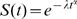
(3)The *density function* is the probability of dying in any interval (here, a particular in-stream age) [28]:
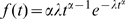
(4)The *hazard function* is then the probability of dying given that the individual survived up to that in-stream age [28]:
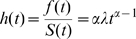
(5)When *α* = 1, the Weibull distribution simplifies to the *exponential* (constant hazard) *distribution*, where hazard is equal to *λ* and does not increase with age (i.e., no senescence). When *α*>1, hazard increases with age and represents true senescence [6], [27], [29].

Because *α* and *λ* are not independent, Ricklefs [26] introduced a derived parameter, *ω* (see also [27], [29]), that provides a shape-adjusted index of the rate of senescence. This parameter has units of time^−1^ and is calculated as:
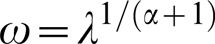
(6)Our primary inferences were based on *ω*, although we also consider *λ* and *α*.

Senescence modeling in *natural* populations requires modifications to the above methods because one must account for individuals whose date of senescent death is not known. In particular, data for tagged individuals include uncensored observations (died of senescence) and censored observations (died of other causes or still alive at the end of the study). When dealing with censored data, the appropriate likelihood function is [28], [41]:
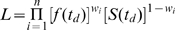
(7)where *f(t)* represents the probability density function, *S(t)* represents the associated survivor function, *t_d_* represents the age at death (in days) of the *i* ^th^ individual, *w* represents the censoring indicator, *i* represents the individual, and *n* represents the total number of individuals. Below, we have substituted the Weibull density and survivor functions into the likelihood function:
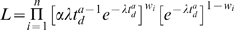
(8)


The censoring indicator can take values of one (uncensored) or zero (censored). For the subset of individuals recovered that died of senescence (i.e., uncensored, *w* = 1), the likelihood function simplifies to the density function, *f(t)*. For individuals that died of other causes (i.e., censored, *w* = 0), the likelihood function simplifies to the survivor function, *S(t)*. Thus, if an individual has died of senescence, we gain information regarding the density function, whereas if an individual has died of other causes, we gain information about the survivor function [28], [41]. The total negative likelihood (*NLL*) for a given model can then be computed by taking the negative of the likelihoods summed across all individuals.

Some populations had many more censored individuals than others (Table 1), and so we evaluated whether censoring had any influence on estimated senescence rates. We expect any such effect to be quite small because censored data provide little information for estimating senescence (i.e., the censored data contribute far less to the total negative log likelihood than do the uncensored data). For example, when considering our best model (model VII, Table 3), 15% of the *NLL* is due to censored observations whereas the remaining 85% is due to uncensored observations of individuals that died of senescence. Moreover, the very large number of censored individuals in two populations (A and C creeks) was due to a single year (1998) when the study had to be terminated earlier and so many individuals were still alive at the end of the study (Table 1). We confirmed that censoring did not influence our conclusions by removing these data from our analyses after which the total number of individuals still alive at the end of the study in these two populations dropped to 40 (A Creek) and 36 (C Creek). Moreover, the estimated rates of senescence did not change: *ω* values for the six streams were almost perfectly correlated between the two data sets (*r^2^* = of 0.98). In short, the censored data do not influence our conclusions.

### Inter-population variation in senescence

Senescence rates were compared among populations by examining models that included or excluded creek-specific *α* and *λ* parameters (Table 3). These models also included “day of entry” parameters because early breeders senesce slower than late breeders [16]. The “day of entry” factor (*R*) for individual *i* was:
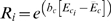
(9)where the subscript *c* indicates creek-specific parameter values, *b_c_* determines how day of entry affects the probability of senescence, 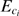 is the day of entry for individual *i* in creek *c*, and *E* *_c_* is the average day of entry for all individuals in the *c* ^th^ creek. In the simplest model, we estimated a single *λ* for all populations. In the most complex model, *λ* was calculated as:
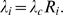
(10)


The fit of alternative models to the data was formally compared based on Akaike's Information Criterion (AIC) [42], [43]:
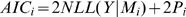
(11)where *NLL* is the negative log likelihood for a given model (*M_i_*) given the data (*Y*), and *P* is the number of parameters in that model. We compared seven models to test for (a) evidence of senescence, (b) the influence of day of entry on senescence, and (c) variation among populations in the rate of senescence. AIC values for our alternative models always differed by at least 15 (Table 3), which indicates much stronger support for the model with the lower AIC value [43].

Finally, we formally tested whether the among-population variation in senescence was better explained by the classic ETS, the condition-dependent ETS, or a combination of the two. To make this assessment, we regressed the shape-adjusted index of senescence (*ω*) against predation rate, predator selectivity, and both factors together (including and excluding their two-way interaction). A positive relationship between the shape-adjusted index of the rate of senescence (*ω*) and predation rate would provide support for the classic ETS, whereas a positive relationship between the rate of senescence and predator selectivity for salmon showing little senescence would provide support for the condition-dependent ETS. We also tested whether variation in senescence was influenced by other environmental factors that might influence the rate of senescence. We did so by regressing *ω* against water temperature, migration distance, and elevation gained during the migration. Interpretations of the relative importance of each factor were made using Akaike's Information Criterion (AIC) to compare models that included or excluded different combinations of the above factors.

## Supporting Information

Text S1 (0.04 MB DOC)

Table S1 Explicit formulae for each of the candidate models for explaining variation in senescence. The α parameter represents the shape of the Weibull hazard function, λ represents the magnitude of the Weibull hazard given its shape, and *R* represents a “day of entry” factor to account for variation in senescence due to variation in day of entry to the breeding grounds. Regardless of parameter, the subscript *c* denotes the *c* ^th^ creek, *i* denotes the *i* ^th^ individual, and the subscript *c^i^* denotes the *i* ^th^ individual in the *c* ^th^ creek. For each model, we present explicit formulae for both the likelihood and the resulting hazard function.
(0.14 MB DOC)

Table S2 Parameter estimates for alternative models. Parameter estimates for α, λ, and ω derived from the second- and third-best models: model V (constant λ, population-specific α values) and model VI (constant α, population-specific λ values), respectively. Variation among populations in ω is here due entirely to variation in α (model V) or variation in λ (model VI). The α parameter represents the shape of the Weibull hazard function, λ represents the magnitude of the Weibull hazard given its shape, and ω is a derived parameter that provides a shape-adjusted index of the rate of senescence [26].
(0.02 MB PDF)

Figure S1 An illustration of the accuracy of our predation rate estimation method. The annual percentage of salmon killed by bears was estimated in each creek based on a single mid-season survey in which the total live and dead (partitioned by mode of death) fish were enumerated. This method was validated by reference to Hansen Creek, where these surveys are performed on each day of the breeding season. Panel A shows the daily predation rate estimates (black circles) calculated as the average of two quantities on that day: (a) the cumulative number of bear-killed salmon divided by the cumulative number of dead salmon (open circles), and (b) the cumulative number of bear-killed salmon divided by the sum of the cumulative number of dead salmon plus the total number of live salmon on that day (grey circles). Note how stable the estimates are over the season and that they closely approximate the actual percentage of bear-killed fish over the entire breeding season (the final points). Panel B shows how a single daily estimate from August 6th each year is highly correlated with the actual percent of salmon killed over the entire breeding season in Hansen Creek (*r^2^* = 0.88; n = 16 years).
(0.43 MB TIF)
