# Supplementary material for: Predation by Bears Drives Senescence in Natural Populations of Salmon
Source: PLoS One. 2007 Dec 12;2(12):e1286. doi: 10.1371/journal.pone.0001286 (PMC3280632; doi:10.1371/journal.pone.0001286)
Supplement: Text S1 — (0.04 MB DOC) [file pone.0001286.s001.doc]

**Text S1.**

We used the method of Gende et al. [21] to model predator selectivity (i.e., condition-dependent mortality) based on the number of salmon of different in-stream ages (*d* days) that were killed by bears. The observed predation rate (*y_d_*) for individuals of a given in-stream age (*d*) was first calculated for each creek as:

(1) $y_{d}=\frac{k_{d}}{a_{d}}$

where $k_{d}$ is the observed number of fish killed by bears of in-stream age of *d*, and $a_{d}$ is the total number of fish of in-stream age of *d* that were available to the bears (i.e., the number of fish that had survived at least *d* days in the stream). The predicted predation rate ($\hat{y}_{d}$) on fish of a given in-stream age of *d* is then:

(2) $\hat{y}_{d}=md+b$

where *m* is the slope and *b* is the intercept of a linear regression between observed predation rates and in-stream age for a given creek. These predicted age-specific predation rates are shown in Figure 2.

As in Gende et al. [21], we employed the negative binomial distribution to calculate the likelihood (*L*) of the predicted number of fish killed by bears given the observed number of kills and the overdispersion parameter, *p*.

(3)

(4) $\Gamma(x)=\int_{0}^{\infty} t^{x-1}e^{-t}dt$

where $k_{d}$ is the observed number of fish killed by bears of in-stream age of *d*, $\hat{k}_{d}$ is the predicted number of fish killed by bears of in-stream age of *d*, and *p* is the overdispersion parameter. The Γ function is another probability density function invoked when using the negative binomial distribution [42].

We next generated for each creek an estimate of overall “predator selectivity” with respect to in-stream age (i.e., state of senescence). This index was designed to avoid potential biases associated with (a) mortality before we were able to observe a fish (in its first day in the creek), and (b) variation among creeks in the availability of fish of different in-stream ages (owing to different rates of extrinsic mortality) [21]. Specifically, we averaged the predicted age-specific predation rates across the first full three days in the stream (i.e., we excluded the day of stream entry). Thus, higher values of predator selectivity indicate stronger selection by bears for fish showing little senescence.
