# Supplementary material for: Predation by Bears Drives Senescence in Natural Populations of Salmon
Source: PLoS One. 2007 Dec 12;2(12):e1286. doi: 10.1371/journal.pone.0001286 (PMC3280632; doi:10.1371/journal.pone.0001286)
Supplement: Table S1 — Explicit formulae for each of the candidate models for explaining variation in senescence. The α parameter represents the shape of the Weibull hazard function, λ represents the magnitude of the Weibull hazard given its shape, and R represents a “day of entry” factor to account for variation in senescence due to variation in day of entry to the breeding grounds. Regardless of parameter, the subscript c denotes the c th creek, i denotes the i th individual, and the subscript ci denotes the i th individual in the c th creek. For each model, we present explicit formulae for both the likelihood and the resulting hazard function. (0.14 MB DOC) [file pone.0001286.s002.doc]

**Table S1**.

|  |  |  |  |  |
| --- | --- | --- | --- | --- |
| Model |  | $\lambda$ | $b$ * | Likelihood |
|  |  |  |  |  |
|  |  |  |  |  |
| I | 1 | $\lambda$ | N/A | $L=\prod_{i=1}^{n} \left[\lambda e^{-\lambda t_{d}}\right]^{w_{i}}\left[e^{-\lambda t_{d}}\right]^{1-w_{i}}$ |
|  |  |  |  |  |
| II |  | $\lambda$ | N/A | $L=\prod_{i=1}^{n} \left[\alpha\lambda t_{d}^{\alpha-1}e^{-\lambda t_{d}^{\alpha}}\right]^{w_{i}}\left[e^{-\lambda t_{d}^{\alpha}}\right]^{1-w_{i}}$ |
|  |  |  |  |  |
| III | $\alpha$ | $\lambda_{i}=\lambda\times R_{i}$ | $b$ | $L=\prod_{i=1}^{n} \left[\alpha\left(\lambda\times R_{i}\right)t_{d}^{\alpha-1}e^{-\left(\lambda\times R_{i}\right)t_{d}^{\alpha}}\right]^{w_{i}}\left[e^{-\left(\lambda\times R_{i}\right)t_{d}^{\alpha}}\right]^{1-w_{i}}$ |
|  |  |  |  |  |
| IV | $\alpha$ | $\lambda_{i}=\lambda\times R_{i}$ | $b_{c}$ | $L=\prod_{i=1}^{n} \left[\alpha\left(\lambda\times R_{i}\right)t_{d}^{\alpha-1}e^{-\left(\lambda\times R_{i}\right)t_{d}^{\alpha}}\right]^{w_{i}}\left[e^{-\left(\lambda\times R_{i}\right)t_{d}^{\alpha}}\right]^{1-w_{i}}$ |
|  |  |  |  |  |
| V | $\alpha_{c}$ | $\lambda_{i}=\lambda\times R_{i}$ | $b_{c}$ | $L=\prod_{i=1}^{n} \left[\alpha_{c}\left(\lambda\times R_{i}\right)t_{d}^{\alpha_{c}-1}e^{-\left(\lambda\times R_{i}\right)t_{d}^{\alpha_{c}}}\right]^{w_{i}}\left[e^{-\left(\lambda\times R_{i}\right)t_{d}^{\alpha_{c}}}\right]^{1-w_{i}}$ |
|  |  |  |  |  |
| VI | $\alpha$ | $\lambda_{i}=\lambda_{c}\times R_{i}$ | $b_{c}$ | $L=\prod_{i=1}^{n} \left[\alpha\left(\lambda_{c}\times R_{i}\right)t_{d}^{\alpha-1}e^{-\left(\lambda_{c}\times R_{i}\right)t_{d}^{\alpha}}\right]^{w_{i}}\left[e^{-\left(\lambda_{c}\times R_{i}\right)t_{d}^{\alpha}}\right]^{1-w_{i}}$ |
|  |  |  |  |  |
| VII | $\alpha_{c}$ | $\lambda_{i}=\lambda_{c}\times R_{i}$ | $b_{c}$ | $L=\prod_{i=1}^{n} \left[\alpha_{c}\left(\lambda_{c}\times R_{i}\right)t_{d}^{\alpha_{c}-1}e^{-\left(\lambda_{c}\times R_{i}\right)t_{d}^{\alpha_{c}}}\right]^{w_{i}}\left[e^{-\left(\lambda_{c}\times R_{i}\right)t_{d}^{\alpha_{c}}}\right]^{1-w_{i}}$ |
|  |  |  |  |  |

* The parameter *b* partly determines variation in *R*, the day of entry parameter, as *b* determines how day of entry affects the probability of senescence. The day of entry parameter was not included in models I and II. In model III, *b* was constrained to be equal among the six populations and so . In models IV – VII, *b* was creek-specific and so.

**Table S2 *continued***

|  |  |  |
| --- | --- | --- |
|  |  |  |
|  | Total no. parameters | Resulting Hazard |
| Model | (parameters) | $h\left(t\right)=\alpha_{c}\lambda_{i}t^{\alpha_{c}-1}$ |
|  |  |  |
|  |  |  |
| I | 1 $\left(\lambda\right)$ | $h\left(t\right)=\lambda$ |
|  |  |  |
| II | 2 $\left(\alpha,\lambda\right)$ | $h\left(t\right)=\alpha\lambda t^{\alpha-1}$ |
|  |  |  |
| III | 3 $\left(\alpha,\lambda,b\right)$ | $h\left(t\right)=\alpha\left(\lambda\times R_{i}\right)t^{\alpha-1}$ |
|  |  |  |
| IV | 8 $\left(\alpha,\lambda,b_{c}\right)$ | $h\left(t\right)=\alpha\left(\lambda\times R_{i}\right)t^{\alpha-1}$ |
|  |  |  |
| V | 13 $\left(\alpha_{c},\lambda,b_{c}\right)$ | $h\left(t\right)=\alpha_{c}\left(\lambda\times R_{i}\right)t^{\alpha_{c}-1}$ |
|  |  |  |
| VI | 13 $\left(\alpha,\lambda_{c},b_{c}\right)$ | $h\left(t\right)=\alpha\left(\lambda_{c}\times R_{i}\right)t^{\alpha-1}$ |
|  |  |  |
| VII | 18 $\left(\alpha_{c},\lambda_{c},b_{c}\right)$ | $h\left(t\right)=\alpha_{c}\left(\lambda_{c}\times R_{i}\right)t^{\alpha_{c}-1}$ |
|  |  |  |
