# Supplementary material for: Predation by Bears Drives Senescence in Natural Populations of Salmon
Source: PLoS One. 2007 Dec 12;2(12):e1286. doi: 10.1371/journal.pone.0001286 (PMC3280632; doi:10.1371/journal.pone.0001286)
Supplement: Table S2 — Parameter estimates for alternative models. Parameter estimates for α, λ, and ω derived from the second- and third-best models: model V (constant λ, population-specific α values) and model VI (constant α, population-specific λ values), respectively. Variation among populations in ω is here due entirely to variation in α (model V) or variation in λ (model VI). The α parameter represents the shape of the Weibull hazard function, λ represents the magnitude of the Weibull hazard given its shape, and ω is a derived parameter that provides a shape-adjusted index of the rate of senescence [26]. (0.02 MB PDF) [file pone.0001286.s003.pdf]

Table S2.

	Model V			Model VI		
Population	*α*	*λ*	*ω*	*α*	*λ*	*ω*
A	4.717	2.66E-06	0.106	4.61	3.54E-06	0.107
Bear	4.383	2.66E-06	0.092	4.61	1.45E-06	0.091
C	4.445	2.66E-06	0.095	4.61	1.7E-06	0.094
Hansen	5.062	2.66E-06	0.120	4.61	8.75E-06	0.125
Pick	4.220	2.66E-06	0.085	4.61	7.99E-07	0.082
Yako	4.710	2.66E-06	0.106	4.61	3.37E-06	0.106
