# Supplementary figures and images for: Predation by Bears Drives Senescence in Natural Populations of Salmon
Source: PLoS One. 2007 Dec 12;2(12):e1286. doi: 10.1371/journal.pone.0001286 (PMC3280632; doi:10.1371/journal.pone.0001286)

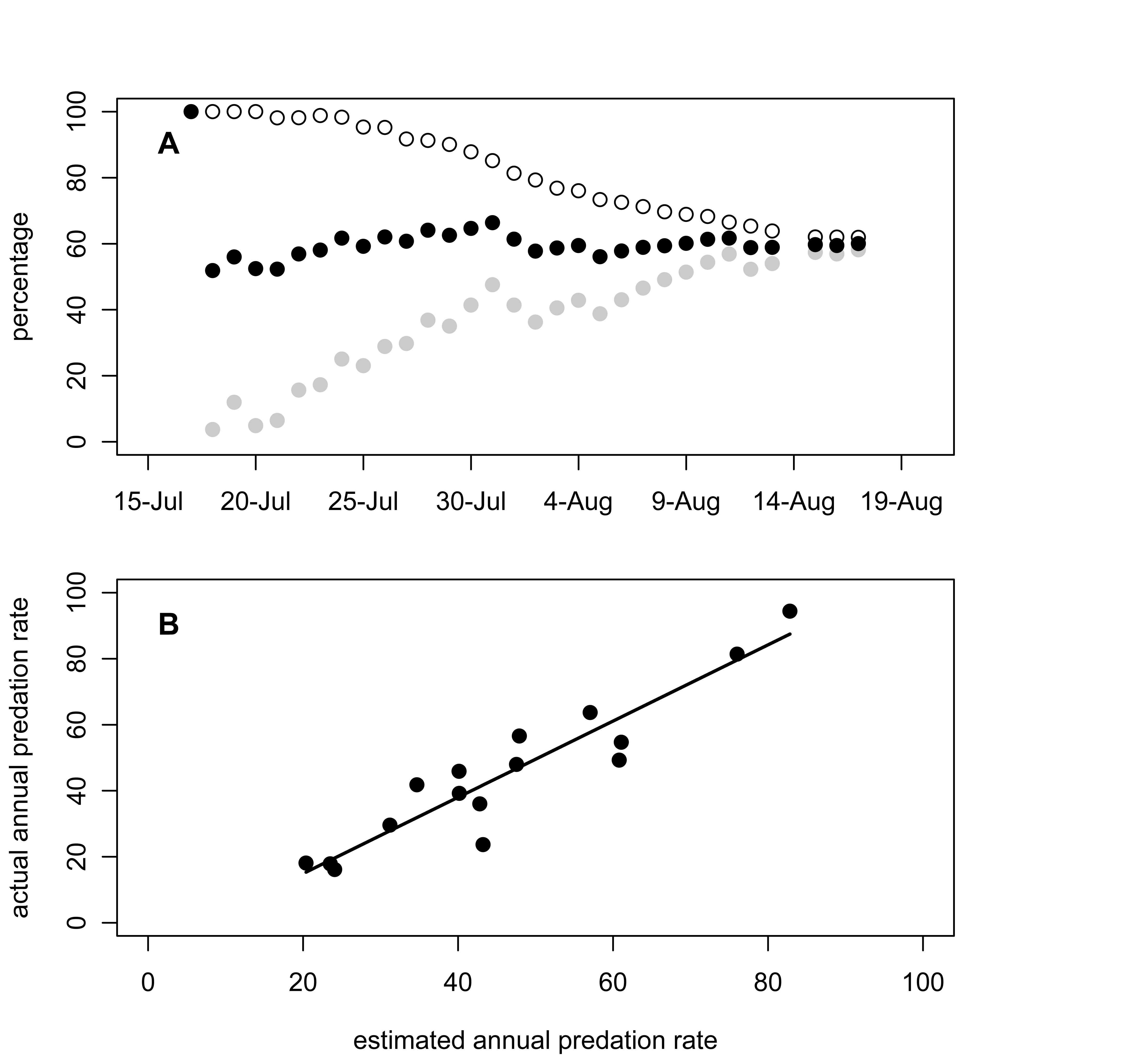

Supplement: Figure S1 — An illustration of the accuracy of our predation rate estimation method. The annual percentage of salmon killed by bears was estimated in each creek based on a single mid-season survey in which the total live and dead (partitioned by mode of death) fish were enumerated. This method was validated by reference to Hansen Creek, where these surveys are performed on each day of the breeding season. Panel A shows the daily predation rate estimates (black circles) calculated as the average of two quantities on that day: (a) the cumulative number of bear-killed salmon divided by the cumulative number of dead salmon (open circles), and (b) the cumulative number of bear-killed salmon divided by the sum of the cumulative number of dead salmon plus the total number of live salmon on that day (grey circles). Note how stable the estimates are over the season and that they closely approximate the actual percentage of bear-killed fish over the entire breeding season (the final points). Panel B shows how a single daily estimate from August 6th each year is highly correlated with the actual percent of salmon killed over the entire breeding season in Hansen Creek (r2 = 0.88; n = 16 years). (0.43 MB TIF) [file pone.0001286.s004.tif]
